# Hospitalisation for bed rest for women with a triplet pregnancy: an abandoned randomised controlled trial and meta-analysis

**DOI:** 10.1186/1471-2393-5-8

**Published:** 2005-04-04

**Authors:** Jodie M Dodd, Caroline A Crowther

**Affiliations:** 1 The University of Adelaide Department of Obstetrics and Gynaecology The Women's and Children's Hospital 72 King William Street North Adelaide South Australia 5006 Australia

## Abstract

**Background:**

This abandoned randomised controlled trial assessed the effects of hospitalisation from 24 to 30 weeks gestation for women with a triplet pregnancy on the risk of preterm birth.

**Methods:**

Women with a triplet pregnancy and no other condition necessitating hospital admission were approached for participation in the study, and randomised to either antenatal hospitalisation (hospitalised group), or to routine antenatal care (control group). The randomisation schedule used variable blocks with stratification by parity, and a researcher not involved with clinical care contacted by telephone to determine treatment allocation by opening the next in a series of consecutively numbered, opaque, sealed envelopes. Primary study outcomes were preterm birth (defined as birth less than 37 weeks gestation) and very preterm birth (defined as birth less than 34 weeks gestation), and the development of maternal pregnancy induced hypertension. The trial was ceased prior to achieving the calculated sample size due to difficulties in recruitment. The results of this randomised controlled trial were then combined with the results of another comparing bed rest in women with a triplet pregnancy.

**Results:**

Seven women with a triplet pregnancy were recruited to the trial, with three randomised to the hospitalisation group, and four to the control group. There were no statistically significant differences between the two groups for the primary outcomes birth before 37 weeks (3/3 hospitalisation group versus 4/4 control group; relative risk (RR) not estimable), birth before 34 weeks (3/3 hospitalisation group versus 2/4 control group; RR 2.00 95% Confidence Intervals (CI) 0.75–5.33) and pregnancy induced hypertension (1/3 hospitalisation group versus 1/4 control group; RR 1.33 95%CI 0.13–13.74).

When the results of this trial were incorporated into a meta-analysis with the previous randomised controlled trial assessing hospitalisation and bed rest for women with a triplet pregnancy, (total sample size 26 women and 78 infants), there were no statistically significant differences identified between the two groups.

**Conclusion:**

The results of this trial and meta-analysis suggest no benefit of routine hospitalisation and bed rest for women with a triplet pregnancy to reduce the risk of preterm birth. The adoption or continuation of a policy of routine hospitalisation and bed rest for women with an uncomplicated triplet pregnancy cannot be recommended.

## Background

Women and infants of a multiple pregnancy are recognised to be at increased risk of adverse outcome when compared with singletons. The greatest risk to infants of a multiple pregnancy is being born preterm, with preterm birth, defined as birth less than 37 weeks gestation, and very preterm birth less than 32 weeks gestation. The preterm birth rate less than 37 weeks for women with a singleton pregnancy is 6.3% versus 97% for women with a triplet pregnancy [1] the mean gestational age of birth for infants of a triplet pregnancy being 31.9 weeks, with 39.3% if infants born before 32 weeks gestation, and a further 57.7% between 32 and 36 weeks gestation [1]. Infants of a triplet pregnancy are at increased risk of poor intrauterine growth, with the mean birth weight of a triplet infant being 1668 grams, compared 3398 with grams in singleton infants [1]. At birth, 15.9% of triplet infants weigh less than 1000 grams, 35.9% less than 1500 grams, and 92.9% less than 2500 grams [1]. Infants of a higher order multiple pregnancy are at increased risk of perinatal death, with a rate of 53.0/1000, almost 7 times greater than that observed in singletons [1].

Several studies have demonstrated a favourable effect of bed rest for women with a triplet pregnancy on fetal growth [2,3]. However, advice regarding the timing and duration of bed rest has varied, including hospitalisation from 28 to 30 weeks gestation until birth [4], from 24 weeks until the beginning of the third trimester [5], or only at the onset of complications [6].

Hospital admission has been advocated in the past for women with a twin pregnancy, as a means of reducing the risk of preterm birth and improving fetal growth [7]. However, the Cochrane Systematic Review assessing the role of hospitalisation and bed rest for women with an uncomplicated twin pregnancy has found the practice to be associated with an increase in the risk of preterm birth, and should not be offered as part of routine care [8].

The value of admission to hospital for rest in triplet or higher order multiple pregnancy is uncertain, with little consistent information available. Several retrospective studies assessing bed rest for women with a triplet pregnancy suggest a reduction in the risk of preterm birth [9,10], while others have not demonstrated a prolongation in gestation [4,11]. The effect of bed rest on perinatal mortality is similarly associated with inconsistent findings, some authors reporting a reduction in mortality [4,9,10], others not [11].

In the only small, randomised study to date in 19 triplet pregnancies, hospitalisation for rest suggests a beneficial trend in reducing the incidence of preterm birth and of increased birthweight in the hospitalised group [12]. All of these beneficial results are compatible with chance variation. There is a need for further evaluation of the effects of admission to hospital for rest in women with a triplet pregnancy.

Any potentially beneficial effects of hospitalisation and bed rest for infant health outcomes must be considered in light of the physical and psychosocial effects on the pregnant woman [13,14]. The separation from family members and the practical issues related to this separation have been identified as a considerable stressor associated with hospitalisation [13].

This randomised controlled trial was designed to assess the effects of hospitalisation from 24 to 30 weeks gestation for women with a triplet pregnancy on the risk of preterm birth. Our primary hypotheses were that routine hospitalisation of women with a triplet pregnancy from 24 to 30 weeks gestation would be associated with a reduction in the incidence of preterm birth (defined as birth less than 37 weeks), and very preterm birth (defined as birth less than 34 weeks).

## Methods

Women with a triplet pregnancy, with ultrasound confirmed gestational age of less than 24 weeks were approached from the antenatal clinic of the Women's and Children's Hospital for participation in the study. Women with a triplet pregnancy and any other condition requiring hospitalisation (for example, placenta praevia) were excluded from participation in the trial. Approval was obtained from the research and ethics committee of the Women's and Children's Hospital. Recruitment commenced in 1996 and was terminated 2003 due to a lack of success in securing research funding to support multicentred collaboration, and difficulties experienced in recruiting sufficient women.

Women with a triplet pregnancy were identified early in their antenatal care, provided with the trial information, and asked to discuss their participation with a family member. Women who provided informed written consent were then randomised to either antenatal hospitalisation (hospitalised group), or to routine antenatal care (control group). The randomisation schedule used variable blocks with stratification by parity, and was prepared by an investigator not involved in clinical care. After completion of the trial entry details, an independent researcher responsible for treatment allocation was contacted by telephone, and the next in a series of consecutively numbered, opaque, sealed envelopes opened. The treatment allocation was stated over the telephone for either hospitalisation for bed rest (hospitalised group) or not (control group) according to the instructions enclosed in the envelope.

Women allocated to the hospitalised group were admitted to hospital from 24 weeks gestation until 30 weeks gestation, after which time, women were discharged home and encouraged to obtain as much rest as possible. All women were able to ambulate within the hospital, received a normal hospital diet, and fortnightly routine antenatal assessments. Women were allowed leave from the ward over weekend periods to assist with compliance with continued hospitalisation.

Women allocated to the control group were encouraged to continue with their normal activities at home, and were reviewed fortnightly in the antenatal clinic. They were admitted to hospital if any complications developed, such as preterm labour, preterm prelabour ruptured membranes, and pregnancy induced hypertension.

Baseline characteristic were obtained to describe the two groups at randomisation, and included maternal age, booking weight and height, smoking and alcohol use, mode of conception (spontaneous conception versus conception via assisted reproductive techniques), previous pregnancy outcomes, and use of antenatal corticosteroids for fetal lung maturation. The primary study outcomes were the incidence of preterm birth (defined as birth less than 37 weeks gestation) and very preterm birth (defined as birth less than 34 weeks gestation), and the development of maternal pregnancy induced hypertension (defined as blood pressure greater than 140/90 mmHg or an increase in the diastolic blood pressure of more than 15 mmHg from booking. Secondary outcome measures included tocolytic use, mode of birth, infant Apgar score of less than seven at five minutes, infant birth weight less than 2500 grams, infant birth weight less than 1500 grams, admission to the neonatal unit, length of stay in the neonatal unit more than seven days, perinatal death (stillbirth and neonatal death), and the occurrence of neonatal morbidity (including respiratory distress syndrome, intraventricular haemorrhage, and necrotising enterocolitis).

The previous randomised controlled trial assessing hospitalisation and bed rest for women with a triplet pregnancy suggests a reduction in the occurrence of preterm birth less than 34 weeks gestation from 44% to 30% [12]. A sample of 400 women would be able to detect this difference, at a level of statistical significance of 5%, and power of 80%. Using preterm birth less than 37 weeks gestation, a sample of 52 women would be able to detect a reduction from 100% to 80% (p = 0.05; power = 80%).

Baseline characteristics were compared, to assess comparability of the treatment groups at trial entry. All randomised women were included in the analysis on an intention to treat basis. Relative risks and 95% confidence intervals were calculated for primary and secondary outcomes. These results were then incorporated into a systematic review and meta-analysis with the previous trial of hospitalisation and bed rest for women with a triplet pregnancy [12].

## Results

Seven women with a triplet pregnancy at less that 24 weeks gestation were recruited to the trial, with three women randomised to the hospitalisation group, and four women to the control group. Baseline characteristics between the two groups were comparable (Table 1).

**Table 1:** Baseline antenatal characteristics

**Characteristic**	**Hospitalisation Group N = 3**	**Control Group N = 4**
Gestational age at randomisation (weeks)*	23.4 ± 1.7	22.0 ± 1.8
Maternal Age (years)*	33.2 ± 0.8	36.2 ± 13.2
Booking Weight (kg)*	62.3 ± 11.0	61.3 ± 3.2
Height (cm)*	170.0 ± 1.5	164.5 ± 3.5
Caucasian	3	4
Married / Defacto	3	4
Primigravid	2	2
Smoker	1	0
Alcohol use in pregnancy	1	0
Conception spontaneous	1	2

*mean and standard deviation

Of the women in the hospitalisation group, two developed antenatal complications (one pregnancy induced hypertension, and one recurrent antepartum haemorrhage), two gave birth by caesarean section (both prelabour procedures), and two developed postnatal complications (one primary postpartum haemorrhage, and one raised blood pressure). All three women were administered antenatal corticosteroids, but none were administered antenatal tocolytic agents. All three women gave birth at less than 37 weeks gestation, and two at less than 34 weeks gestation (Table 2).

**Table 2:** Primary outcome measures

**Outcome**	**Hospitalisation Group N = 3**	**Control Group N = 4**	**Relative Risk**	**95% CI**
Birth <37 weeks gestation	3	4	Not estimable	
Birth <34 weeks gestation	3	2	2.00	0.75 to 5.33
Pregnancy induced hypertension	1	1	1.33	0.13 to 13.74

Figures are numbers

Of the women in the control group, three were admitted during their antenatal course (one for threatened preterm labour, one for pregnancy induced hypertension, and one for recurrent antepartum haemorrhage and one for intrauterine growth restriction), three of the four women were administered antenatal corticosteroids and one woman received antenatal tocolytic agents. All four women gave birth by caesarean section (three elective prelabour procedures), and three developed postnatal complications (one postpartum haemorrhage and two raised blood pressure). All four women gave birth at less than 37 weeks gestation, and two women gave birth at less than 34 weeks gestation. There were no statistically significant differences between the two groups for any of the infant outcomes (Table 3). There were no stillbirths and two neonatal deaths in one triplet grouping in the control group (one due to tracheal stenosis and the other to necrotising enterocolitis and bowel infarction).

**Table 3:** Secondary outcome measures

**Outcome**	**Hospitalisation Group N = 9**	**Control Group N = 12**	**Relative Risk**	**95% CI**
Gestational age at birth (weeks)*	33.5 ± 2.7	33.5 ± 3.5	-0.01	-4.60 to 4.53
Birth weight (grams)*	1892 ± 251.8	1810 ± 551.8	82.00	-314.28 to 478.28
Birth weight <2500 grams	9	8	1.12	0.89 to 1.42
Birth weight <1500 grams	1	3	0.33	0.04 to 2.63
Placental weight (grams)*	906.7 ± 136.5	1210.5 ± 557.9	-303.83	-1082.18 to 474.52
Apgar score <7 at 5 mins	0	1	0.33	0.04 to 2.63
Neonatal Death	0	2	0.20	0.01 to 3.66
Neonatal Morbidity	0	1	0.33	0.02 to 7.24

Figures are numbers and relative risk or *mean and standard deviation, and weighted mean difference

When the results of this trial are incorporated into a meta-analysis with the previous randomised controlled trial assessing hospitalisation and bed rest for women with a triplet pregnancy [12], a total sample size of 26 women and 78 infants is obtained. There were no statistically significant differences identified between the two groups for the outcomes reported (Table 4).

**Table 4:** Meta-analysis of two randomised trials assessing bed rest for triplets

**Outcome**	**Number Trials**	**Number Participants**	**Relative Risk**	**95% Confidence Intervals**
Gestational age at birth (weeks)*	2	26	0.57	-1.36 to 2.51
Birth <37 weeks gestation	2	26	0.80	0.59 to 1.09
Birth <34 weeks gestation	2	26	0.87	0.36 to 2.08
Caesarean section	2	26	1.18	0.47 to 2.96
Maternal hypertension	2	26	0.43	0.11 to 1.72
Birth weight <2500 grams	2	75	0.87	0.36 to 2.08
Birth weight <1500 grams	2	75	1.18	0.47 to 2.96
Apgar score <7 at 5 mins	2	75	0.43	0.11 to 1.72
Neonatal unit admission	2	71	0.90	0.74 to 1.09
Neonatal stay >7 days	2	71	1.39	0.80 to 2.42
Perinatal Death	2	78	2.71	0.12 to 63.84
Neonatal Death	2	75	0.19	0.02 to 1.53

*weighted mean difference

## Discussion

The results of this small randomised trial and meta-analysis suggest no benefit of routine hospitalisation and bed rest for women with a triplet pregnancy in reducing the risk of preterm birth and improving fetal growth. The effect of the additional seven women recruited from this trial has been to reduce the magnitude of potential beneficial trends identified previously [12], with the point estimates for outcomes coming closer to unity. While the combined sample size in the meta-analysis of 26 women remains underpowered to be able to reliably detect differences in rates of preterm birth and in particular, perinatal mortality, it is unlikely that there will be further attempts to answer the question of the value of hospitalisation and bed rest in women with triplet pregnancies in the form of randomised controlled trials. In any case, the reduction in magnitude of potential benefits somewhat reduces the degree of uncertainty that remains in clinical practice.

The difficulties encountered in recruitment to this trial highlight a number of issues. Randomised trials where the target population (in this case, women with a triplet pregnancy) comprise a small proportion of the obstetric population will, by necessity, require multicentred collaboration. While this trial received ethics approval from a number of collaborating centres, all women recruited were from the coordinating centre. This may reflect an inability of busy clinicians to actively recruit women due to time constraints within their practice, or lack of specific funding, thereby relying on collaborators to act altruistically, with little financial or other rewards for multicentre collaboration [15]. While the current health care system may not be conducive to active participation in clinical research, it is unlikely that the structure will change sufficiently in the short term. Another equally difficult approach may be to change the attitude of obstetricians, with greater emphasis, particularly during training, on the need for research. If clinical research is then viewed as a "normal" component of clinical practice, participation and recruitment to clinical trials may be facilitated [15]. After all, in the face of uncertainty about a clinical intervention or treatment, the most ethical response on the part of the clinician is to offer participation in a clinical trial [16], which essentially involves "choice under uncertainty, plus data collection" [17].

## Conclusion

The results of this trial and meta-analysis suggest no benefit of routine hospitalisation and bed rest for women with a triplet pregnancy in terms of reducing the risk of preterm birth and improving fetal growth. The adoption or continuation of a policy of routine hospitalisation and bed rest for women with an uncomplicated triplet pregnancy cannot be recommended.

## Competing interests

The author(s) declare that they have no competing interests.

## Authors' contributions

CAC conceived the study and was involved in the study coordination, data analysis and revisions of the manuscript. JMD was involved in verification of the data, data analysis, drafted the original manuscript and contributed to subsequent revision. Both authors read and approved the final manuscript.

## Pre-publication history

The pre-publication history for this paper can be accessed here:


